# Supplementary material for: Mitochondrial protein import determines lifespan through metabolic reprogramming and de novo serine biosynthesis
Source: Nat Commun. 2022 Feb 3;13:651. doi: 10.1038/s41467-022-28272-1 (PMC8814026; doi:10.1038/s41467-022-28272-1)

SUPPLEMENTARY INFORMATION

Mitochondrial protein import determines lifespan through metabolic reprogramming and *de novo* serine biosynthesis

Eirini Lionaki[1,α]*, Ilias Gkikas[1,2,α], Ioanna Daskalaki[1,2], Maria-Konstantina Ioannidi[3,4], Maria I. Klapa[3] & Nektarios Tavernarakis[1,5]*

[1]Institute of Molecular Biology and Biotechnology, Foundation for Research and Technology - Hellas, [2]Department of Biology, School of Sciences and Engineering, University of Crete, [3]Metabolic Engineering and Systems Biology Laboratory, Institute of Chemical Engineering Sciences, Foundation for Research and Technology-Hellas (FORTH/ICE-HT), Patras, Greece; [4]Department of Biology, University of Patras, Patras, Greece; [5]Department of Basic Sciences, Faculty of Medicine, University of Crete, Heraklion 71110, Crete, Greece.

*Correspondence and requests for materials should be addressed to E.L. (e-mail: lionaki@imbb.forth.gr), or to N.T. (e-mail: tavernarakis@imbb.forth.gr).

CONTENTS

1. Supplementary Figures 1-10
2. Supplementary Table 1
3. Supplementary Table 2
4. Supplementary Table 3
5. Images of blots included in Supplementary Figure 3e

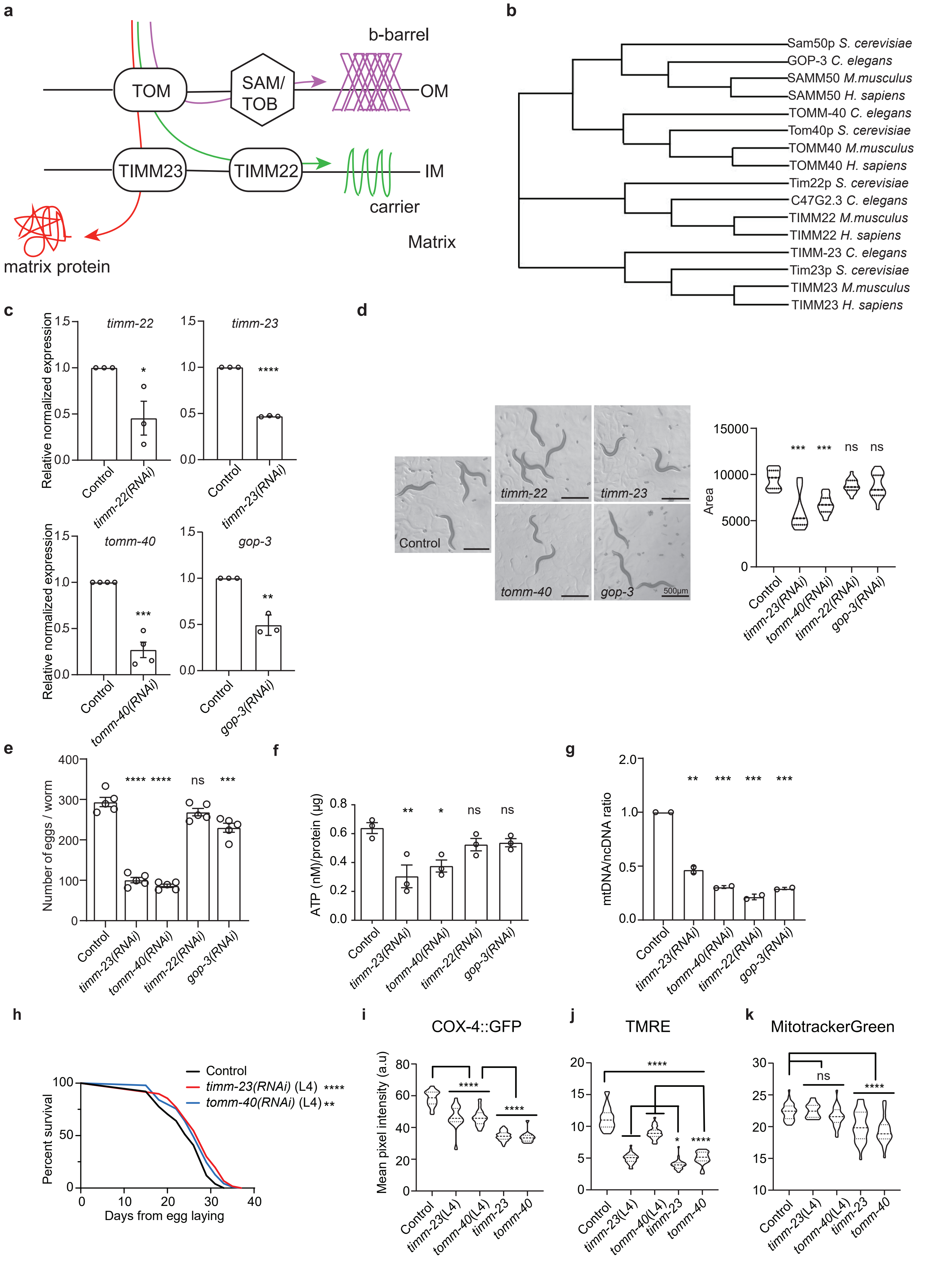

Supplementary Figure 1

Supplementary Figure 1. Phenotypic characterization of animals with mitochondrial protein import deficiencies. a Schematic diagram of the basic protein import routes to mitochondria. The localization and role of each translocase (TOM, TIMM23, TIMM22, TOB/SAB) is indicated. **b** Protein sequences obtained through Blast search in different species were used for the phylogenetic tree, generated in Clustal Omega (https://www.ebi.ac.uk/Tools/msa/clustalo). **c** Relative expression of *timm-22*, *timm-23*, *tomm-40* and *gop-3* mRNA levels upon genetic inhibition of each of them. n=3 biologically independent experiments, two-tailed *t*-test was used for comparisons. Data are presented as mean expression ±SEM. **d** Animal body size upon genetic inhibition of *timm-22*, *timm-23*, *tomm-40* and *gop-3* with the Image J software (Area). n=3 biologically independent experiments, mean area ±SEM One-way ANOVA with Dunnett's multiple comparison test. Exact sample size and P values are included in Source Data file. (**** denotes *P<0.0001* and ns denotes not significant) **e** Brood size of animals treated with *timm-22*, *timm-23*, *tomm-40* and *gop-3* RNAi. n=5 biological independent experiments **f** Total ATP content per μg of protein of RNAi treated wt worms on their first day of adulthood. Data presented as mean values ±SEM. n=3 biologically independent, One-way ANOVA Dunnett's multiple comparison test. Exact P values are included in Source Data file (** denotes P<0.01 and * denotes P<0.05) **g** Quantification of the relative mtDNA copy number of animals treated with *timm-22*, *timm-23*, *tomm-40* and *gop-3* RNAi. n=2, two-tailed *t*-test was used for comparisons. Data presented as mean expression ±SD. **h** Lifespan curves of animals treated with *timm-23* and *tomm-40* RNAi from the L4 stage. n=2 biologically independent experiments. Curves were compared with the Log-rank (Mantel-Cox) test (**** denotes P<0.0001, ** denotes P<0.001); detailed values are shown in Supplementary Table 2. **i-k** Quantified fluorescence of COX-4::GFP reporter animals (**i**), mitochondrial

membrane Δψ (TMRE stained animals) (**j**) and total mitochondrial mass (Mitotracker Green stained animals) (**k**) upon treatment with *timm-23* and *tomm-40* RNAi from the L4 stage and from egg. n=3 biologically independent experiments. One-way ANOVA, Tukey's multiple comparisons test (**** denotes $P<0.0001$, * denotes $P<0.05$, ns denotes not significant). Exact sample size and P values are included in Source Data file. a.u.: arbitrary units.

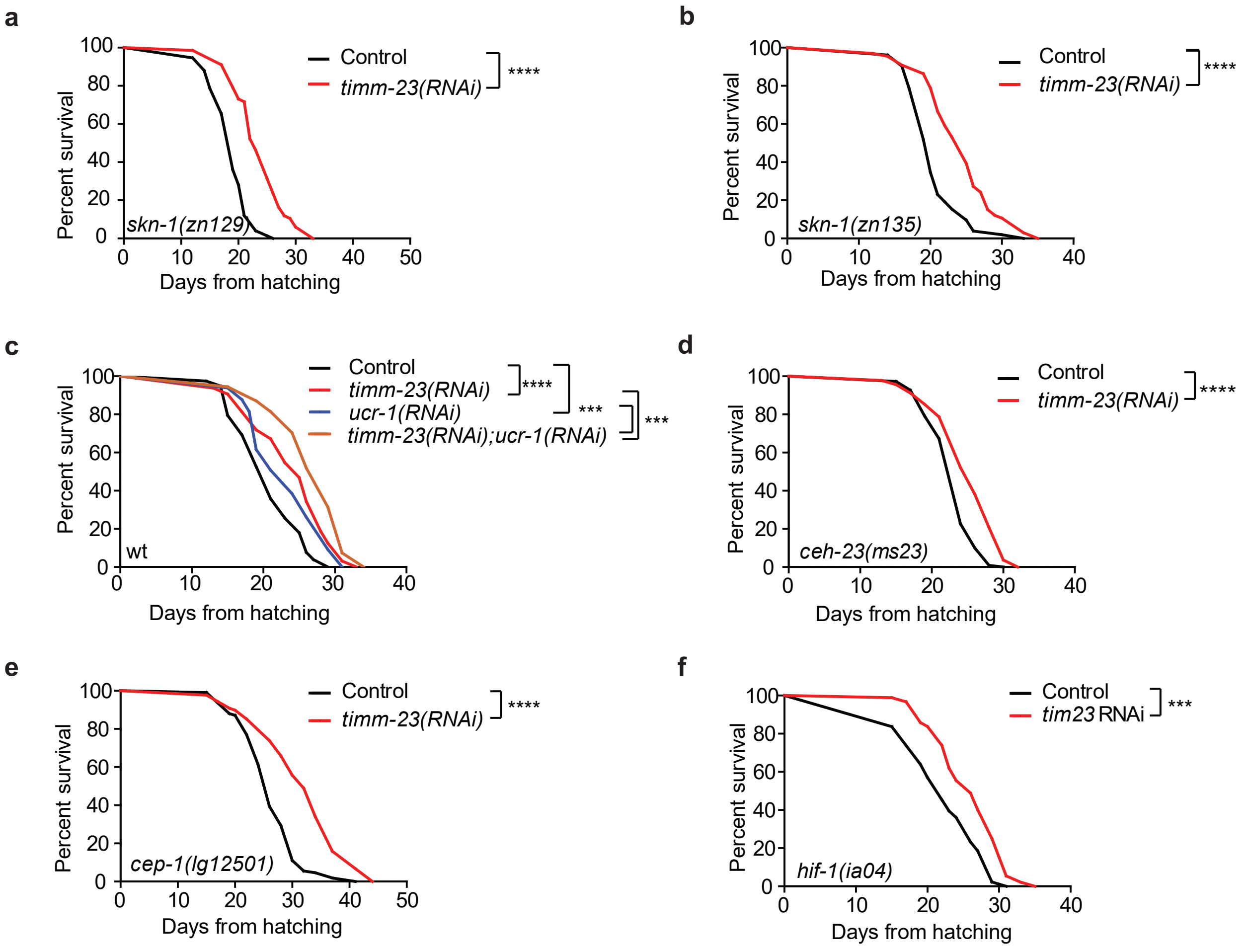

Supplementary Figure 2

Supplementary Figure 2. MitoMISS extends lifespan independently of well-characterised longevity pathways. a-f Lifespan curves of well-characterized longevity mutants such as *skn-1(zn129)* (a), *skn-1(zn135)* (**b**), *ucr-1* respiration-deficient animals (**c**), *ceh-23(ms23)* (**d**), *cep-1(lg12501)* (**e**), and *hif-1(ia04)* (**f**) upon MitoMISS are indicated. Survival curves were compared with the Log-rank (Mantel-Cox) test (*** denotes $P<0.0001$, ** denotes $P<0.001$); detailed values are shown in Supplementary Table 2.

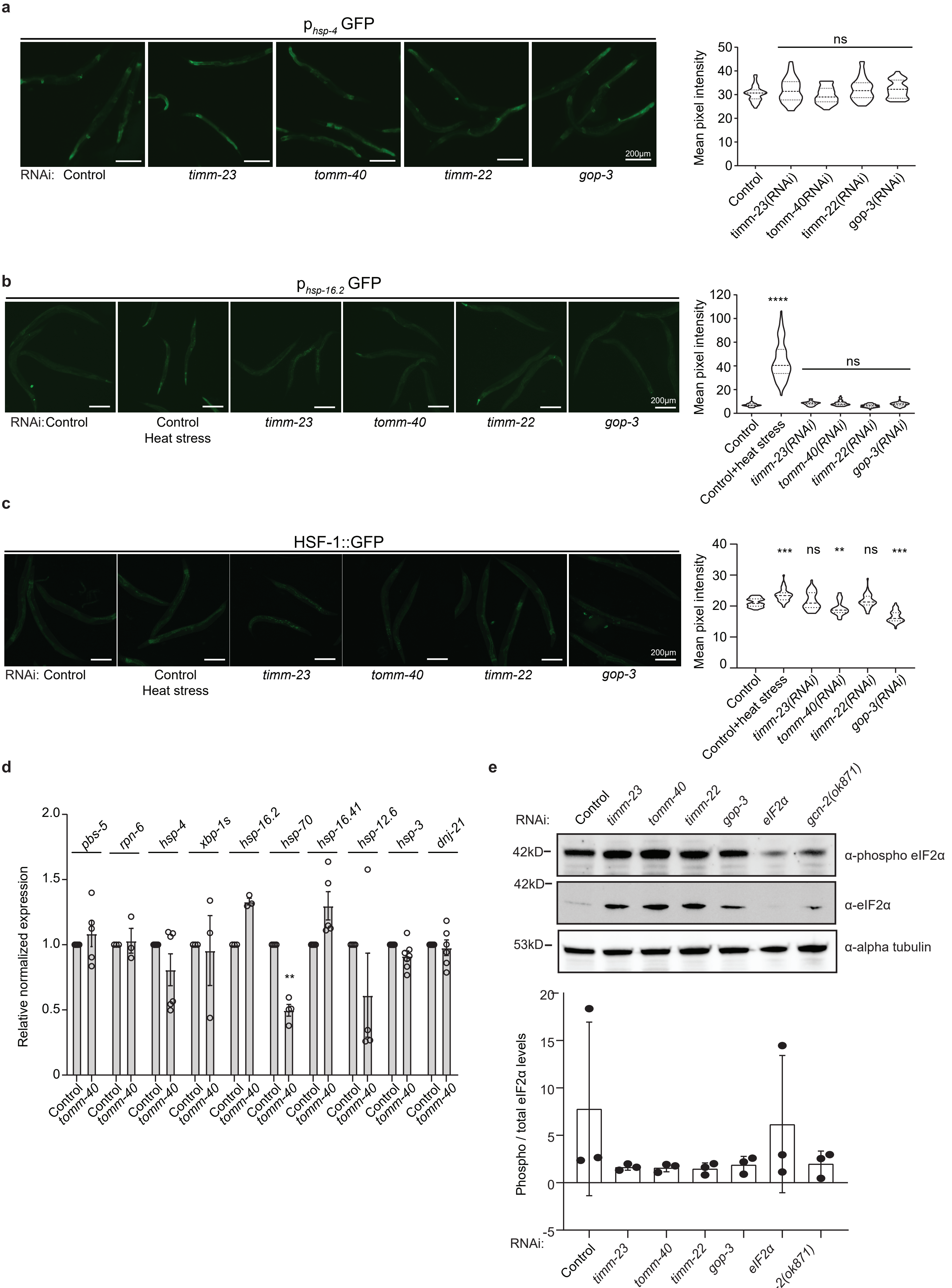

Supplementary Figure 3

Supplementary Figure 3. Association of MitoMISS with various proteostatic networks. a-c Epifluorescence images of *hsp-4* transcript levels (UPR^{ER}) (**a**), *hsp-16.2* transcript levels (HSR) (**b**) and HSF-1 protein levels (**c**) upon *timm-23*, *tomm-40*, *timm-22* and *gop-3* genetic inhibition (left panels) and their corresponding quantifications depicted in violin plots (right panels), one-way ANOVA, with Dunnett's multiple comparisons test. n=3 biologically independent experiment with at least 20 worms per condition. Exact sample size and P values are included in Source Data file. a.u.: arbitrary units **d** Normalised expression levels of proteasomal (*pbs-5* and *rpn-6*), UPR^{ER} (*hsp-4* and *xbp-1s*) and heat stress response marker genes (*hsp-16.2*, *hsp-70* (C12C8.1), *hsp-16.41*, *hsp-12.6*, *hsp*-3, *dnj-21*), relative to the housekeeping genes *pmp-3* and *act-3* (n≥3, normalised mean expression ±SEM, 2way ANOVA, with Sidak's multiple comparisons test). **e** Immunoblot analysis of the phosphorylated and the total protein levels of eIF2α upon *timm-23*, *tomm-40*, *timm-22* and *gop-3* genetic inhibition (**** denotes $P<0.0001$, *** denotes P<0.001, ** denotes P<0.01 and * denotes P<0.05). Quantified ration of phospho/total eIF2α levels from three independent experiments (Image J software) are shown in the bar graph below as mean ±SD.

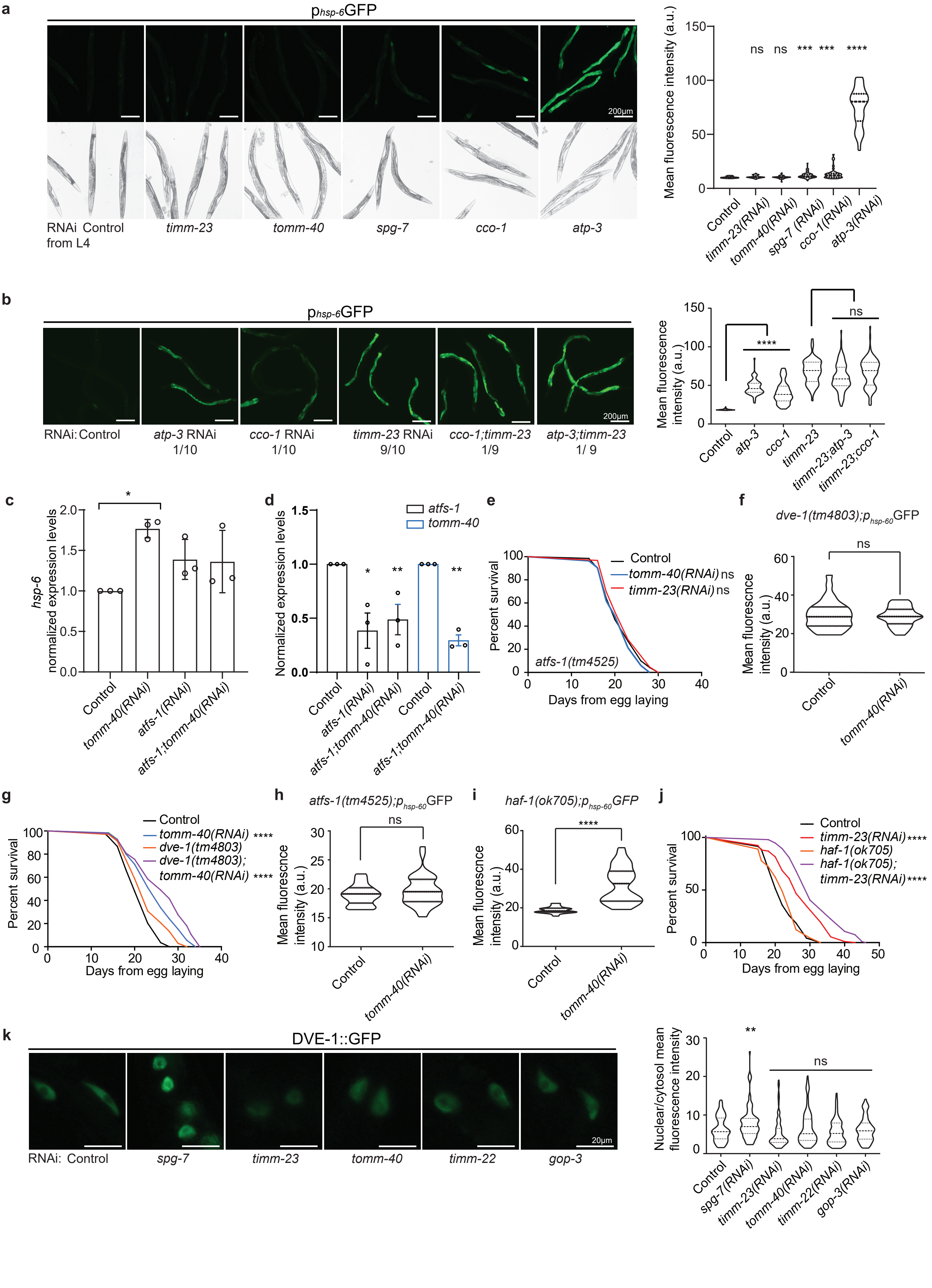

Supplementary Figure 4

Supplementary Figure 4. Components of canonical UPR^{mt} are not consistently activated or required for longevity upon MitoMISS. a Epifluorescence images of *hsp-6* transcriptional reporter upon *timm-23*, *tomm-40*, *spg-7, cco-1* and *atp-3* genetic inhibition performed at the L4 stage (left panel) and the corresponding quantification (right panel) n=2 biologically independent experiments with at least 20 worms per condition. Two-tailed *t*-test. Exact sample size and P values are included in Source Data file. **b** Epifluorescence images of *hsp-6* transcript levels upon 10% *cco-1* and *atp-3,* 90% *timm-23* and their combination performed from egg (left panel) and the corresponding quantification of $p_{hsp\text{-}6}$GFP fluorescence (right panel) n=2 biologically independent experiments with at least 20 worms per condition. One-way ANOVA, with Tukey's multiple comparison test). Exact sample size and P values are included in Source Data file. **c** Relative expression of *hsp-6* mRNA in *tomm-40*, *atfs-1* and *atfs-1;tomm-40* RNAi treated animals. n=3 biologically independent experiments.one-way ANOVA with Dunnett's multiple comparison test **d** Relative expression of *atfs-1* mRNA in *atfs-1* and *atfs-1;tomm-40* RNAi treated animals and of *tomm-40* mRNA in *atfs-1;tomm-40* RNAi treated animals. n≥2 Data presented as normalised mean expression ±SD **e** Lifespan curves upon MitoMISS in the absence of ATFS-1. n=2 biologically independent experiments with at least 20 worms per condition. **f** Fluorescence intensity of $p_{hsp\text{-}60}$GFP transgenic worms crossed with *dve-1(tm4803)*. *tomm-40* RNAi does not increase *hsp-60* expression in the *dve-1(tm4803)* mutant background. n=3 biologically independent experiments with at least 20 worms per condition. Exact sample size and P values are included in Source Data file. **g** Survival curves of *tomm-40 RNAi* extends lifespan of the *dve-1(tm4803)* mutant. n=2 biologically independent experiments. **h-i** Fluorescent intensity of $p_{hsp\text{-}60}$GFP transgenics crossed with *atfs-1(tm4525)* (**h**) or *haf-1(ok705)* (**i**) mutants. n=2

biologically independent experiments with at least 20 worms per condition. Comparison with two-tailed *t*-test. Exact sample size and P values are included in Source Data file. (**j**) MitoMISS potently increases lifespan of *haf-1(ok705)* mutants. n=2 biologically independent experiments. **k** Nuclear/cytoplasmic mean fluorescent intensity of DVE-1::GFP. Two-tailed *t*-test (** denotes P<0.01 and ns denotes not significant). n=2 biologically independent experiments with at least 20 worms per condition. Exact sample size and P values are included in Source Data file. Survival curves were compared with the Log-rank (Mantel-Cox) test (*** denotes P<0.0001, ** denotes P<0.001) ; detailed values are shown in Supplementary Table 2. a.u.: arbitrary units.

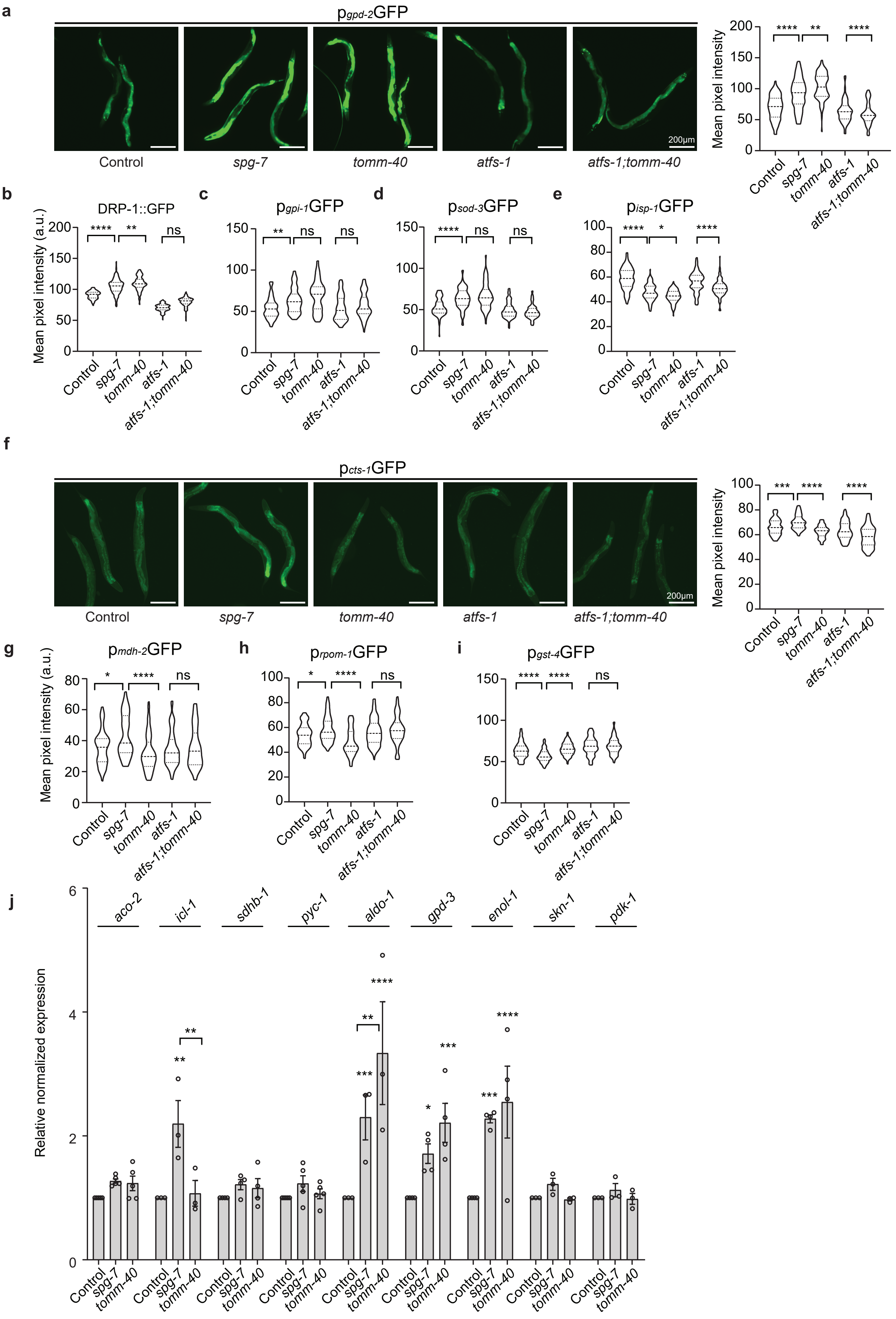

Supplementary Figure 5

Supplementary Figure 5. Shared and differential expression patterns of known ATFS-1 target genes upon MitoMISS. a Epifluorescence images showing the expression levels of *gpd-2* transcript, upon inhibition of *spg-7*, *tomm-40*, *atfs-1* and *atfs-1;tomm-40* (left panel) and the corresponding quantification (right panel). **b** Quantification of DRP-1::GFP fluorescence upon inhibition of *spg-7*, *tomm-40*, *atfs-1* and *atfs-1;tomm-40*. **c-e** Quantified fluorescence of the *gpi-1*(**c**), *sod-3* (**d**) and *isp-1* (**e**) transcriptional reporters upon inhibition of *spg-7*, *tomm-40*, *atfs-1* and *atfs-1;tomm-40.* **f** Epifluorescence images of the *cts-1* transcriptional reporter, upon inhibition of *spg-7*, *tomm-40*, *atfs-1* and *atfs-1;tomm-40* (left panel) and the corresponding quantification (right panel). **g-i** Quantified fluorescence of the *mdh-2* (**g**), *rpom-1* (**h**) and *gst-4* (**i**) transcriptional reporters upon inhibition of *spg-7*, *tomm-40*, *atfs-1* and *atfs-1;tomm-40.* Quantified fluorescence intensities are depicted in violin plots. N≥2 biologically independent experiments of at least 20 worms per condition. One-way ANOVA, with Tukey's multiple comparisons test (**** denotes *P<0.0001*, *** denotes P<0.001, ** denotes P<0.01 and * denotes P<0.05). Exact sample size and P values are included in Source Data file. a.u.: arbitrary units (**j**) Normalized expression levels of the indicated ATFS-1 targets genes, relative to the housekeeping genes *pmp-3* and *act-3* (n≥3, ±SEM, two-way ANOVA, with Sidak's multiple comparisons test).

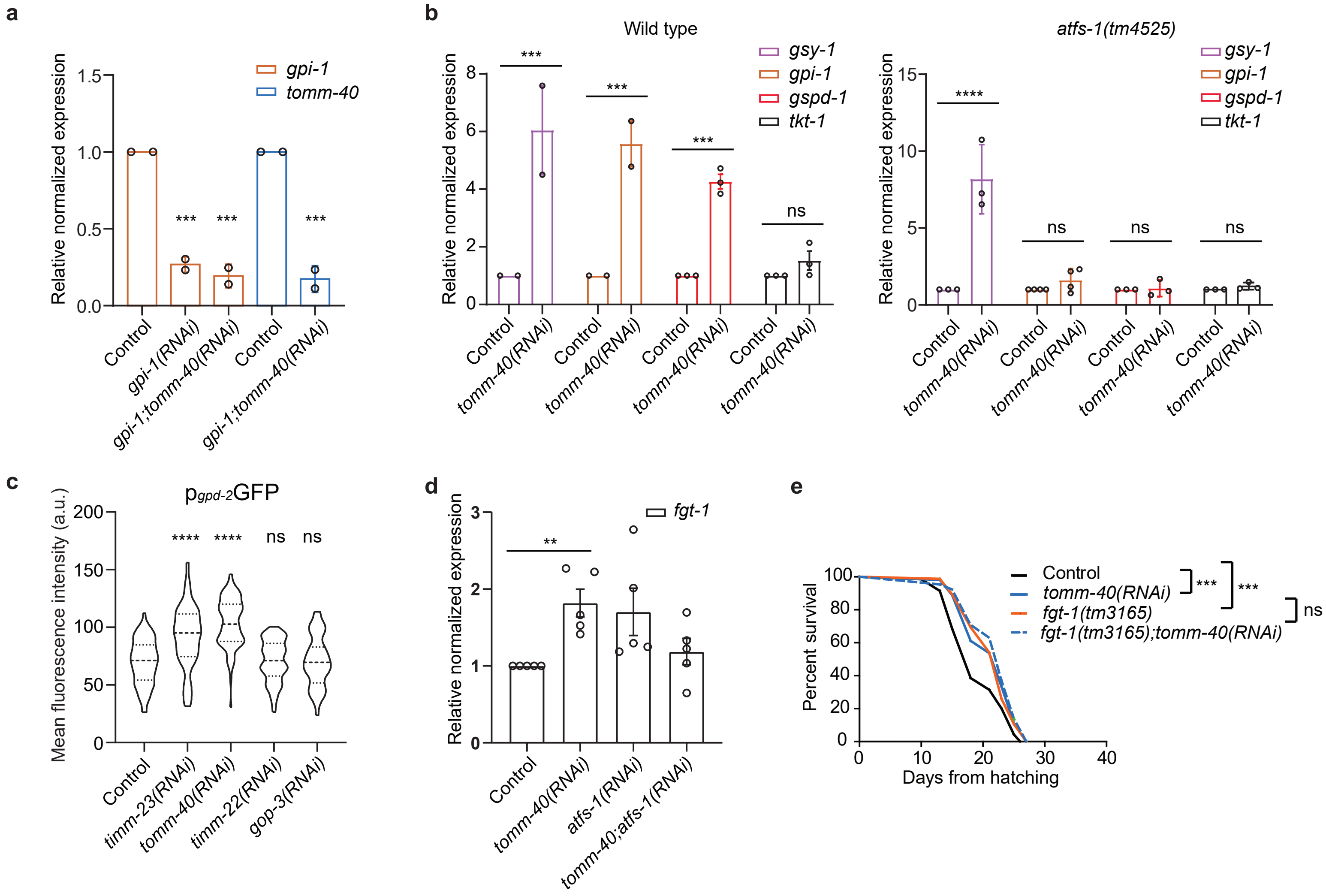

Supplementary figure 6

Supplementary Figure 6. MitoMISS activates expression of genes involved in glucose metabolism. a Relative expression of *gpi-1* mRNA in *gpi-1* and *gpi-1;tomm-40* RNAi treated animals and of *tomm-40* mRNA in *gpi-1;tomm-40* RNAi treated animals. **b** Relative expression of *gsy-1*(grey), *gpi-1*(green), *gspd-1*(pink) and *tkt-1*(blue) mRNA in control and *tomm-40* RNAi treated wild type animals (left panel) and *atfs-1* mutants (right panel). Expression levels were normalized to housekeeping genes *act-3* and *pmp-3*. N=2 biologically independent experiments. Mean expression ±SD. Two-way ANOVA Sidak's multiple comparisons test. **c** Expression levels of the glycolysis transcriptional reporter, *gpd-2,* upon MitoMISS, *timm-22* and *gop-3* inhibition, n=3 biologically independent experiments (one-way ANOVA, with Tukey's multiple comparisons test. Exact sample size and P values in Source Data file **d** Relative expression of *fgt-1* mRNA in *tomm-40*, *atfs-1* and *atfs-1;tomm-40* RNAi treated animals. n=5 biological independent experiments. Mean expression ±SEM. Comparisons with two-tailed *t*-test. **e** Lifespan of wt or *fgt-1(tm3165)* mutants upon MitoMISS. n=2 biological independent experiments. Statistical analysis of survival curves was performed with the Log-rank (Mantel-Cox) test (*** denotes *P<0.0001*, ** denotes P<0.001 and * denotes P<0.01); detailed values are shown in Table S2. a.u.: arbitrary units.

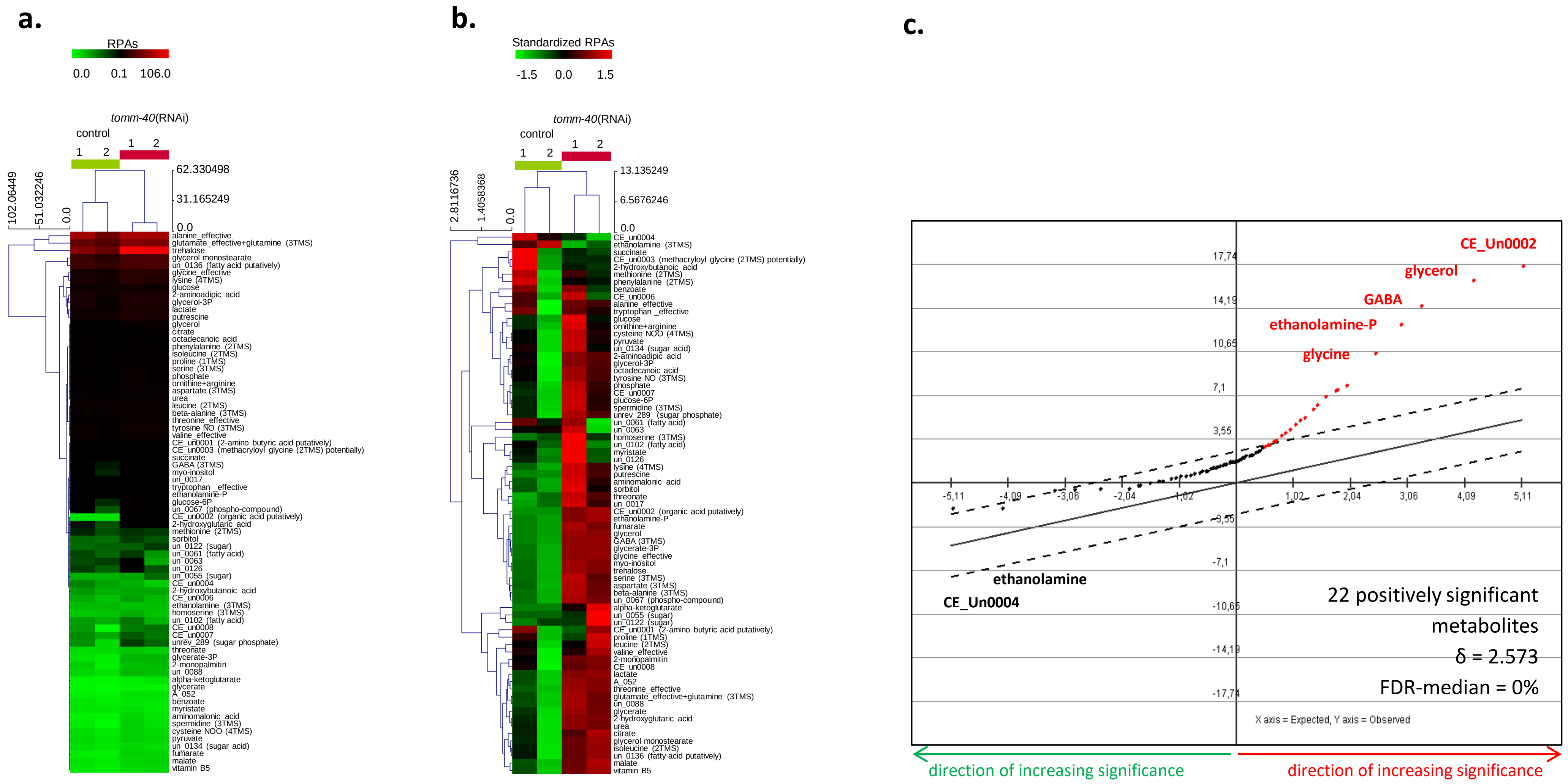

Supplementary Figure 7

Supplementary Figure 7: Metabolic profiling of control and MitoMISS animals- Collection time 1 (CT1). a Hierarchical clustering analysis of the normalized metabolomic dataset for collection time 1 (CT1) based on Euclidean distance (median RPA = 1.27). It depicts the difference in the abundance between the various metabolites with trehalose being among the most abundant with a clear increase in the *tomm-40* RNAi worms compared to the controls; **b** Hierarchical clustering analysis of the standardized metabolic profiles for collection time 1 (CT1) based on Euclidean distance. The metabolites of differential abundance between the control and *tomm-40* RNAi worms are apparent; **c** The SAM curve of the metabolic profile data of the *tomm-40* RNAi worms (Group B) compared to the controls (Group A). Each point corresponds to a particular metabolite; axes correspond to the observed (y axis) and the expected (x axis) values calculated by the method for each metabolite. The expected value concerns the case in which the difference in a metabolite abundance between the two groups would have been based on random error. The dashed lines define the smallest threshold of significance δ for which FDR (median) is equal to zero. If the difference between the measured and the expected value for a metabolite abundance is in absolute value larger than δ, then if positive or negative, this metabolite is, respectively, identified of significantly higher (red) or lower (green) abundance in the B compared to the A group. The further a significant metabolite is from the origin (0,0), the higher the statistical significance of the metabolite. The full list of positive, significant metabolites are shown in Supplementary Table 3 and their abundance is also depicted in the heat map of Fig. 5a and b. Here indicated are the 8 positively significant metabolites exhibiting the largest difference in Group B vs A and the two exhibiting the largest decrease in Group B vs A, which is, however, not identified as significant in the context of the overall

increase in the total 73 metabolite abundance (positive intercept with y axis) in tomm40 RNAi worms compared to the controls.

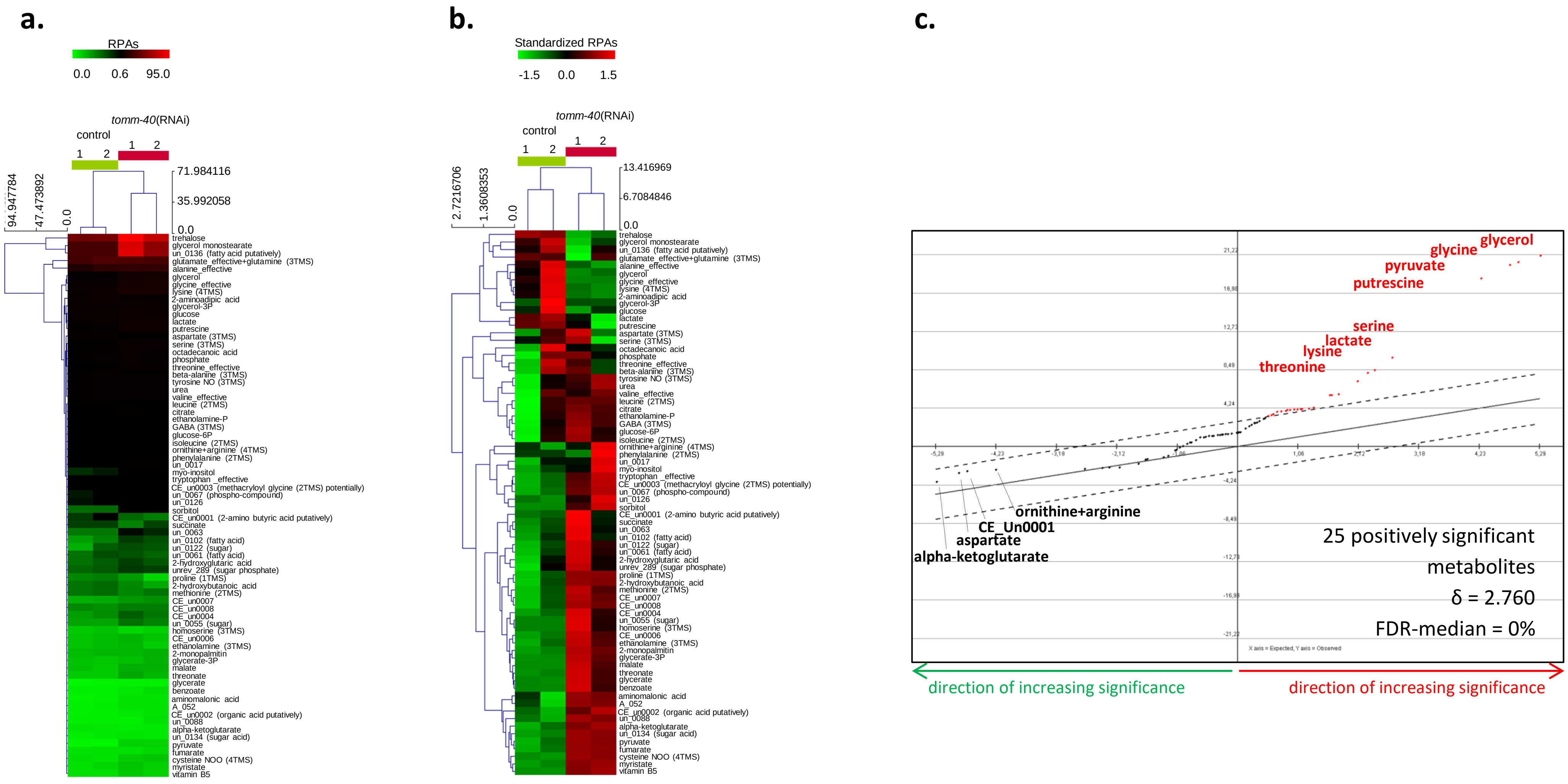

Supplementary Figure 8

Supplementary Figure 8: Metabolic profiling of control and MitoMISS animals-Collection time 2 (CT2). a Hierarchical clustering analysis of the normalized metabolomic dataset for collection time 2 (CT2) based on Euclidean distance (median RPA = 0.6). It depicts the difference in the abundance between the various metabolites with trehalose being the most abundant and with a clear increase in the *tomm-40* RNAi worms compared to the controls; **b** Hierarchical clustering analysis of the standardized metabolic profiles for collection time 2 (CT2) based on Euclidean distance. The metabolites of differential abundance between the control and *tomm-40* RNAi worms are apparent; **c** The SAM curve of the metabolic profile data of the *tomm-40* RNAi worms (Group B) compared to the controls (Group A). Details of the graph are as described in Supplementary Table 3. The full list of positively significant metabolites are shown in Supplementary Table 3 and their abundance is also depicted in the heat map of Figure 5a and b. Here indicated are the 8 positively significant metabolites exhibiting the largest difference in Group B vs. A, and the four exhibiting the largest decrease in Group B vs. A, but are not identified as significant in the context of the overall increase in the total 72 metabolite abundance (positive intercept with y axis) in *tomm-40* RNAi worms compared to the controls.

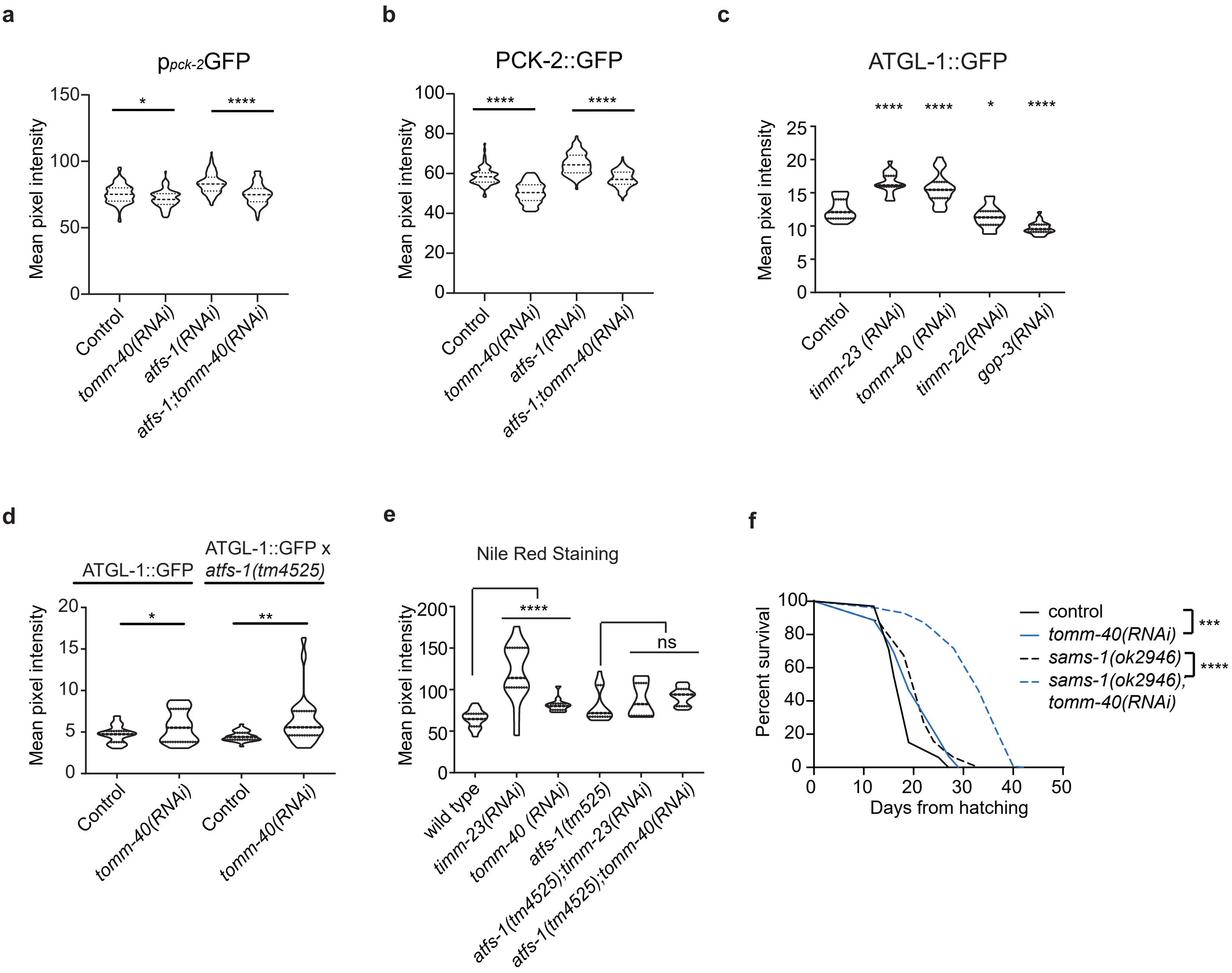

Supplementary figure 9

Supplementary Figure 9. MitoMISS induces fat mobilization and extends lifespan of *sams-1* mutants. **a** Fluorescence intensity of *pck-2* transcriptional reporter or **b** translationa reporter under control, *tomm-40*, *atfs-1* and *atfs-1;tomm-40* RNAi. n=3 biologically independent experiments with at least 20 worms per condition. One-way ANOVA with Tukey's multiple comparisons test. Exact sample size and P values in Source data file. **c** Fluorescence intensity of the translation reporter worms VS20 expressing full length ATGL-1 under its endogenous promoter, treated with the indicated RNAi constructs from hatching until day 1 of adulthood. n=3 biologically independent experiments with at least 20 worms per condition. Two-tailed *t*- test. Exact sample size and P values in Source data file. **d** VS20 worms crossed with *atfs-1(tm4525)* mutants and treated with the indicated RNAi constructs from hatching until day 1. n=3 biologically independent experiments with at least 20 worms per condition. Two-tailed *t*- test. Exact sample size and P values in Source data file. **e** Total fluorescence intensity of wild type worms treated with the indicated RNAi constructs from hatching until L4 and dyed after fixation with the lipid-specific dye Nile Red. n=3 biologically independent experiments with at least 10 worms per condition. Two-tailed *t*- test. Exact sample size and P values in Source data file. a.u.: arbitrary units **f** Lifespan analysis of *sams-1(ok2946)* mutant animals upon MitoMISS. n=2 biological independent experiments. Survival curves were compared with the Log-rank (Mantel-Cox) test (**** denotes $P<0.0001$) ; detailed values are shown in Supplementary Table 2. a.u. : arbitrary units .

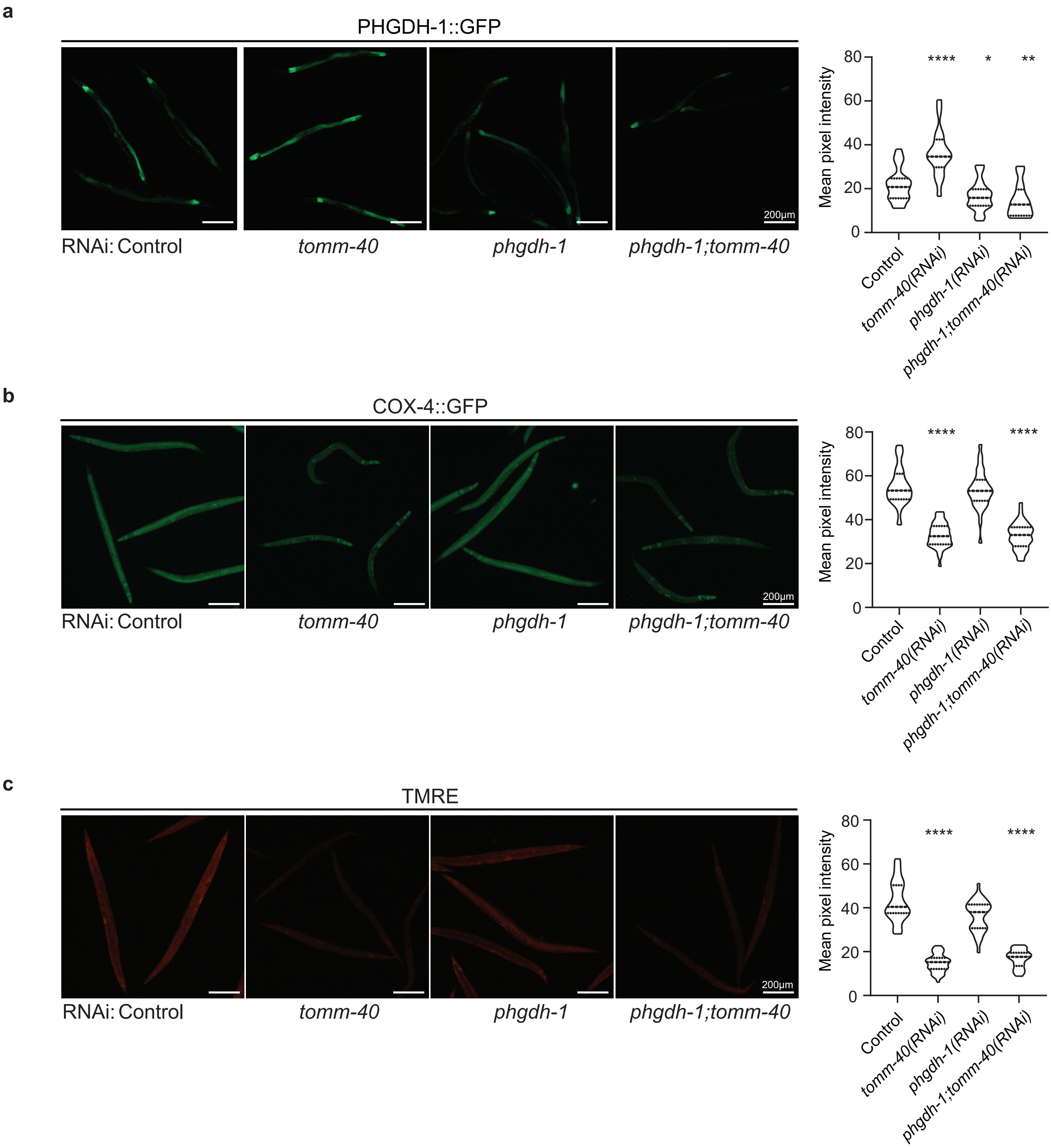

Supplementary figure 10

Supplementary Figure 10. Inhibition of PHGDH-1 does not affect mitochondrial abundance and function. a-c Fluorescence intensity of *phgdh-1* translational reporter (**a**), COX-4::GFP reporter (**b**) or TMRE stained wild type worms (**c**) treated with control, *tomm-40*, *phgdh-1* and *phgdh-1;tomm-40* RNAi. Fluorescent intensity of different samples was compared to that of the control sample with one-way ANOVA with Dunnett's multiple comparison. n=3 biological independent experiments with at least 15 worms per condition. Exact sample size and P values in Source Data file(**** denotes $P<0.0001$, *** denotes P<0.001 and ** denotes P<0.01). a.u.: arbitrary units

Supplementary Table 1: Summary of oligonucleotides used in the present study.

Gene	Sequence	Enzymes for subcloning	Target vector
timm-22 RNAi Forward	ATGGCGCTGGCCTCCTCAAATG	HindIII-XbaI	pL4440
timm-22 RNAi Reverse	ACCGGTGGTCCTCTCATATAATGATCAATAATTG	HindIII-XbaI	pL4440
timm-23 RNAi Forward	ATGGGTTGGTTCGGATTC	HindIII-NotI	pL4440
timm-23 RNAi Reverse	CACTGTCAGTTTGTGTTACCA	HindIII-NotI	pL4440
tomm-40 RNAi Forward	ATGGCGACACCAACAGAAAGTGAG	EcoRI	pL4440
tomm-40 RNAi Reverse	ACCGGTGGACCAATGATAAGTCCG	EcoRI	pL4440
gop-3 RNAi Forward	ATGTCTGAAAAGACGTTCCACAAG	BamHI-NotI	pL4440
gop-3 RNAi Reverse	CTACAAGAAGTTGACACCAGCTCC	BamHI-NotI	pL4440
atfs-1 RNAi Forward	ATGTTTTCCCGTGTGGGACG	KpnI-XbaI	pL4440
atfs-1 RNAi Reverse	TCATCGAGTTGATCTCACGCTTTAG	KpnI-XbaI	pL4440
gpi-1 RNAi Forward	CCATGTCTCTGTCTCAAGACGCC	KpnI-ApaI	pL4440
gpi-1 RNAi Reverse	GAAATCTACCTGATGATGCAATCCC	KpnI-ApaI	pL4440
phgdh-1 RNAi Forward	ATGAGCGCCCCAATCAACAAAGTC	KpnI-ApaI	pL4440
phgdh-1 RNAi Reverse	GTTGAGCACCTCTCGTGCGTTCAAAA	KpnI-ApaI	pL4440
dve-1 RNAi Forward	GAGCTCATGTTCCCAATGAGGG	XbaI-SpeI	pL4440
dve-1 RNAi Reverse	GAGCTCGAAAACCTTCTGCTCAAG	XbaI-SpeI	pL4440
fgt-1 promoter Forward	TCTCCAAGTCTCATGGTGCATC	BamHI-AgeI	pPD96.75
fgt-1 promoterReverse	ACCGGTGACGGAATCTAAGTAGTATTAGTGGGG	BamHI-AgeI	pPD96.75
hsp-60 RNAi Forward	CTTTCCGCACGATCATTCTTG	EcoRI	pL4440
hsp-60 RNAi Reverse	GGTTCCACGATCTCTGGCTG	EcoRI	pL4440
phgdh-1 promoter Forward	CTAGATTCCACCAAATTGGCAGATTGC	BamHI-AgeI	pPD96.75
phgdh-1 coding region Reverse no stop	CCCGGGGGGTTGAGCACCTCTCG	BamHI-AgeI	pPD96.75
Glifon4000 Forward	GGATCCATGGTGAGCAAGGGCGAGGAGCT	BamHI-EcoRI	pPD96.75
Glifon4000 Reverse	GAATTCCGAATTACTTGTACAGCTCGTCCATGC	BamHI-EcoRI	pPD96.75
spg-7 RNAi Forward	CCAGGAGGATGGCAACAAATTG		pL4440
spg-7 RNAi Reverse	AGAGTGCATATCCGCCGCCA		pL4440
atp-3 RNAi Forward	AGAACAAGCTCGACCAGATTTC		pL4440
atp-3 RNAi Reverse	GGGCATCCTTGTATTTCTTGAC		pL4440
cco-1 RNAi Forward	GATGGCTCAACTTGCTAAGACG		pL4440
cco-1 RNAi Reverse	CATCTCTTTGGATCTCCTTTGC		pL4440
act-3 RT Forward	ATCCGTAAGGACTTGTACGCCAAC	-	For qPCR
act-3 RT Reverse	CGATGATCTTGATCTTCATGGTTC	-	For qPCR
pmp-3 RT Forward	CTTGCTGGAGTCACTCATCGTGTTATG	-	For qPCR
pmp-3 RT Reverse	GTCGGGACGCTGATTTATCATCTTC	-	For qPCR
timm-23 RT Forward	GATTCGATGCTCCAGCGCC	-	For qPCR
timm-23 RT Reverse	TGAGGGATCCATCTGCACATAG	-	For qPCR
tomm-40 RT Forward	CAAGCCACAATTGAGAGAAAAGG	-	For qPCR
tomm-40 RT Reverse	CCATACTGATATACCATCTCAGTTCC	-	For qPCR
timm-22 RT Forward	GAGGAGATCCTACGAAACAGCTAAC	-	For qPCR
timm-22 RT Reverse	GACAGCAAAACCAGCGGC	-	For qPCR
gop-3 RT Forward	CACAGTATACATATACTGTAAAGGGCG	-	For qPCR
gop-3 RT Reverse	CGCATTCAATTTGACTTGATGC	-	For qPCR
gpi-1 RT Forward	GCTAGATTCGAGAAATTTACCCG	-	For qPCR
gpi-1 RT Reverse	GCATGACGTCCTTTCCGTC	-	For qPCR
fgt-1 RT Forward	GTGCCCAGAGTCTCCAAAGTAC	-	For qPCR
fgt-1 RT Reverse	CTCCCTTGAACATATCTCCCATT	-	For qPCR
gsy-1 RT Forward	GAATCTCTCTGGTTTCGGATGC	-	For qPCR

gsy-1 RT Reverse	GCGGCGGCAGTCACG	-	For qPCR
icl-1 RT Forward	GCTTCGAGTTGATGAAGGCC	-	For qPCR
icl-1 RT Reverse	GGTTCTTGCGACAATGATTGT	-	For qPCR
tkt-1 RT Forward	ACGTCTTCTACGTCTGCTGCC	-	For qPCR
tkt-1 RT Reverse	GATGAAGTTCAAGCGAGGAGTG	-	For qPCR
hsp-6 RT Forward	GAACCGGAAAGGAACAACAGATCG	-	For qPCR
hsp-6 RT Reverse	GCAATCTTGGTTCGGAGAGCCTC	-	For qPCR
gspd-1 RT Forward	GAAGTGCTCATACGTTCAGGG	-	For qPCR
gspd-1 RT Reverse	CCGAGATAATGATCAATACGGTAG	-	For qPCR
pdk-1 RT Forward	GAAGGAGCCTACAGCCAGGTATTC	-	For qPCR
pdk-1 RT Reverse	TAGCCTGGTCGTGAAAATGTGTG	-	For qPCR
skn-1 RT Forward	TCCACCAGGATCTCCATTCG	-	For qPCR
skn-1 RT Reverse	CTCCATAGCACATCAATCAAGTCG	-	For qPCR
aldo-1 RT Forward	CAAGAAAGACGGAGCACAGTTCG	-	For qPCR
aldo-1 RT Reverse	GTTCACCGTCTGGGAGGATCTCTG	-	For qPCR
gpd-3 RT Forward	CCAGCACAAGATCAAGGTCTAC	-	For qPCR
gpd-3 RT Reverse	CAGATGGAGCAGAGATGATGACCTTC	-	For qPCR
enol-1 RT Forward	GGTTGATCTTTTCACTGAGAAAGGAGTC	-	For qPCR
enol-1 RT Reverse	CTGGAGCGATCTTCTCGTTGATG	-	For qPCR
pyc-1 RT Forward	CGTGGAGAACGCGAAGTCTTC	-	For qPCR
pyc-1 RT Reverse	GGTTGCTTCTTGGTAACCTTGTCG	-	For qPCR
aco-2 RT Forward	AAGATTTGGCCCGTGCTAAGGAC	-	For qPCR
aco-2 RT Reverse	TGAGAAGAAGTCCTGGGAAAGCGTAG	-	For qPCR
sdhb-1 RT Forward	GCCAACTGTGCAGAAATTCG	-	For qPCR
sdhb-1 RT Reverse	CTTCACAACGAACATATGTGGAAG	-	For qPCR
pbs-5 RT Forward	GTCGCCAAGTACTGCACTTTG	-	For qPCR
pbs-5 RT Reverse	CCTGAGCCAACTGAGCACAC	-	For qPCR
rpn-6 RT Forward	CTCAGGCTCTTCCACTTGCTG	-	For qPCR
rpn-6 RT Reverse	AGAGCTGCTTGCATGCGAG	-	For qPCR
hsp-4-4 RT Forward	ACGACCACAATCGTCTCAGTCC	-	For qPCR
hsp-4 RT Reverse	CTTCGTCAGTGAGCTTTCCTCC	-	For qPCR
xbp-1s RT Forward	TGCCTTTGAATCAGCAGTGG	-	For qPCR
xbp-1s RT Reverse	ACCGTCTGCTCCTTCCTCAATG	-	For qPCR
hsp-16.2 RT Forward	TCTCTCCATCTGAGTCTTCTGAGATTG	-	For qPCR
hsp-16.2 RT Reverse	CTACCTGAAGATGTAGATGTTGGTGC	-	For qPCR
dnj-21 RT Forward	GAGGTTTAATTGTTGCCGG	-	For qPCR
dnj-21 RT Reverse	CCTCCTCTATCTGGATGATTTAC	-	For qPCR
hsp-70 RT Forward	ATGTGAACGTGCTAAGCGT	-	For qPCR
hsp-70 RT Reverse	GGTCCTTCCCATTGAAAAAC	-	For qPCR
hsp-16.41 RT Forward	TTCCGATAATATTGGGGAGATTG	-	For qPCR
hsp-16.41 RT Reverse	CGTTTCAAGTATCCATGTTCCG	-	For qPCR
hsp-12.6 RT Forward	GGAGATGGAGTTGTCAATGTCCTC	-	For qPCR
hsp-12.6 RT Reverse	GAATTCCATGTGAATCCAAGTTGC	-	For qPCR
hsp-3 RT Forward	TCCGGTGAGGTCGAACTTT	-	For qPCR
hsp-3 RT Reverse	ACCGTCACCATCCAGGTC	-	For qPCR
atp-6 RT Forward	GTTTATGCTGCTGTAGCGTG	-	For mtDNA qPCR
atp-6 RT Forward	CTGTTAAAGCAAGTGGACGAG	-	For mtDNA qPCR

Supplementary Table 2: Summary of lifespan experiments under all conditions tested.

Strain name	RNAi treatment	Supplement	Median Lifespan from hatching	Number of deaths (n)	Significance	Ref. Control
wt	control	-	23	104		
wt	*timm-23*	-	27	94	****	wt
wt	*timm-40*	-	27	106	****	wt
wt	*timm-23*	-	23	124	ns	wt
wt	*gop-3*		23	9	ns	wt
wt	control	-	23	93		
wt	*gop-3*	-	23	89	ns	wt
wt	*timm-23*	-	25	86	****	wt
wt	*tomm-40*	-	27	73	****	wt
wt	*timm-22*	-	21	99	**	wt
wt	control	-	21	69		
wt	*timm-22*	-	20	101	**	wt
daf-2(e1370)	control	-	48	125		
daf-2(e1370)	*timm-23*	-	53	116	****	*daf-2(e1370)*
daf-2(e1370)	control	-	39.5	60		
daf-2(e1370)	*timm-23*	-	45	136	***	*daf-2(e1370)*
daf-16(mu86)	control	-	20	115		
daf-16(mu86)	*timm-23*	-	27	132	****	*daf-16(mu86)*
daf-16(mu86)	control	-	15	75		
daf-16(mu86)	*tomm-40*	-	21	40	****	*daf-16(mu86)*
eat-2(ad465)	control	-	24	115		
eat-2(ad465)	*timm-23*	-	28	142	****	*eat-2(ad465)*
eat-2(ad465)	control	-	24	79		
eat-2(ad465)	*timm-23*	-	27	86	****	*eat-2(ad465)*
skn-1(zn129)	control	-	20	31		
skn-1(zn129)	*timm-23*	-	25	24	****	*skn-1(zn129)*
skn-1(zn129)	control	-	19	75		
skn-1(zn129)	*timm-23*	-	23	67	****	*skn-1(zn129)*
skn-1(zn135)	control	-	20	52		
skn-1(zn135)	*timm-23*	-	25	66	****	*skn-1(zn135)*
skn-1(zn67)	control	-	19	58		
skn-1(zn67)	*timm-23*	-	20	79	****	*skn-1(zn67)*
wt	control	-	18	147		
wt	*timm-23*	-	20	154	****	wt
aak-2(ok524)	control	-	16	111	**	wt
aak-2(ok524	*timm-23*	-	24	111	****	*aak-2(ok524*

aak-2(ok524)	control	-	20	83		
aak-2(ok524	*timm-23*	-	22	128	****	*aak-2(ok524*
hif-1(ia04)	control	-	22	99		
hif-1(ia04)	*timm-23*	-	28	72	****	*hif-1(ia04)*
hif-1(ia04)	control	-	22	86		
hif-1(ia04)	*timm-23*	-	26	92	****	*hif-1(ia04)*
ceh-23(ms23)	control	-	24	110		
ceh-23(ms23)	*timm-23*	-	26	139	****	*ceh-23(ms23)*
cep-1(lg12501)	control	-	22	118		
cep-1(lg12501)	*timm-23*	-	27	97	****	*cep-1 (lg12501)*
wt	control	-	24	109		
wt	*timm-23*	-	32	103	****	wt
cep-1(lg12501)	control	-	26	109		
cep-1(lg12501)	*timm-23*	-	32	88	****	*cep-1 (lg12501)*
cep-1(gk138)	control	-	24	72		
cep-1(gk138)	*timm-23*	-	26	107	****	*cep-1(gk138)*
ceh-23(ms23)	control	-	22	113		
ceh-23(ms23)	*timm-23*	-	25	131	****	*ceh-23(ms23)*
cep-1(lg12501)	control	-	22			
cep-1(lg12501)	*timm-23*	-	27			
atfs-1(tm4525)	control	-	21	106		
atfs-1(tm4525)	*timm-23*	-	20	101	ns	*atfs-1(tm4525)*
wt	control	-	19	68		
wt	*tomm-40*		20	101	*	wt
atfs-1(tm4525)	control	-	18	90		
atfs-1(tm4525)	*timm-23*	-	16	94	ns	*atfs-1(tm4525)*
atfs-1(tm4525)	*tomm-40*	-	16	87	ns	*atfs-1(tm4525)*
wt	control		21	67		
wt	*timm-23*		24	91	****	wt
wt	*tomm-40*		24	40	****	wt
atfs-1(tm4525)	control	-	20	70		
atfs-1(tm4525)	*timm-23*	-	20	58	ns	*atfs-1(tm4525)*
atfs-1(tm4525)	*tomm-40*	-	20	84	ns	*atfs-1(tm4525)*
wt	control	-	21	69		
wt	*tomm-40*	-	25	71	****	control
wt	*atfs-1*	-	21	57	ns	control
wt	*atfs-1; tomm-40*	-	21	87	ns	*atfs-1*
wt	control	-	23	88		
wt	*timm-23 ½*	-	26	85	****	wt
wt	*atfs-1 ½*	-	23	96	ns	wt

wt	*atfs-1 ½; timm-23 ½*	-	23	108	ns	wt
wt	control	-	23	85		
wt	*timm-23 ½*	-	26	69	****	wt
wt	*atfs-1 ½*	-	23	84	ns	wt
wt	*atfs-1 ½ ; timm-23 ½*	-	24	99	ns	wt
wt	control	-	18	70		
wt	*tomm-40*	-	23	54	***	wt
fgt-1(tm3165)	control	-	23	85	***	wt
fgt-1(tm3165)	*tomm-40*	-	23	65	ns	*fgt-1(tm3165)*
fgt-1(tm3165)	control	-	21	82		
fgt-1(tm3165)	*tomm-40*	-	21	64	ns	*fgt-1(tm3165)*
wt	control	-	22	71		
wt	*tomm-40*	-	30	109	****	wt
Wt	*gpi-1*	-	27	77	****	wt
wt	*gpi-1; tomm-40*	-	22	54	****	*tomm-40*
wt	control	-	22	142		
wt	*tomm-40*	-	26	130	****	wt
Wt	*gpi-1*	-	23	89	**	wt
wt	*gpi-1; tomm-40*	-	21	109	****	*tomm-40*
wt	control	-	21	79		
wt	*tomm-40*	-	24	117	****	wt
wt	*gpi-1*	-	23	162	**	wt
wt	*gpi-1; tomm-40*	-	19	139	****	*tomm-40*
wt	control	-	22	71		
wt	*tomm-40*	-	30	109	****	wt
wt	*phgdh-1*	-	30	27	****	wt
wt	*phgdh-1; tomm-40*	-	22	75	****	*tomm-40*
wt	control	-	21	79		
wt	*tomm-40*	-	24	116	****	wt
wt	*phgdh-1*	-	24	99	****	wt
wt	*phgdh-1; tomm-40*	-	19	106	****	*tomm-40*
wt	control	-	22	142		
wt	*tomm-40*	-	26	130	****	wt
wt	*phgdh-1*	-	23	59	***	wt
	phgdh-1; tomm-40	-	21	114	****	*tomm-40*
wt	control	0mM Serine	18	142		
wt	control	5mM Serine	21	136	****	control (0mM Serine)

wt	control	25mM Serine	18	112	***	control (0mM Serine)
wt	control	50mM Serine	18	113	****	control (0mM Serine)
wt	control	0mM Serine	19	135		
wt	control	5mM Serine	22	125	***	control (0mM Serine)
wt	control	25mM Serine	20	126	**	control (0mM Serine)
wt	control	50mM Serine	20	137	**	control (0mM Serine)
wt	control	0mM Serine	19	126		
wt	control	5mM Serine	23	89	***	control (0mM Serine)
wt	control	25mM Serine	19	131	*	control (0mM Serine)
wt	control	50mM Serine	19	101	**	control (0mM Serine)
wt	*tomm-40*	0mM Serine	22	104		
wt	*tomm-40*	5mM Serine	22	117	ns	*tomm-40* (0mM Serine)
wt	*tomm-40*	25mM Serine	18	112	***	*tomm-40* (0mM Serine)
wt	*tomm-40*	50mM Serine	18	95	***	*tomm-40* (0mM Serine)
wt	*tomm-40*	0mM Serine	23	134		
wt	*tomm-40*	5mM Serine	23	122	ns	*tomm-40* (0mM Serine)
wt	*tomm-40*	25mM Serine	19	120	***	*tomm-40* (0mM Serine)
wt	*tomm-40*	50mM Serine	18	105	***	*tomm-40* (0mM Serine)
wt	*phgdh-1*	0mM Serine	22	114		
wt	*phgdh-1*	5mM Serine	23	151	**	*phgdh-1*(0mM Serine)
wt	*phgdh-1*	25mM Serine	19	109	**	*phgdh-1*(0mM Serine)

wt	*phgdh-1*	50mM Serine	17	116	**	*phgdh-1*(0mM Serine)
wt	*phgdh-1*	0mM Serine	20	126		
wt	*phgdh-1*	5mM Serine	22	71	**	*phgdh-1*(0mM Serine)
wt	*phgdh-1*	25mM Serine	21	93	*	*phgdh-1*(0mM Serine)
wt	*phgdh-1*	50mM Serine	20	117	*	*phgdh-1*(0mM Serine)
wt	control	-	23	109		
wt	*tomm-40*	-	27	112	****	wt
wt	control	5mM Serine	27	120	****	wt
wt	*tomm-40*	5mM Serine	27	89	ns	5mM Serine control
wt	control	5mM Serine	23	80		
wt	*tomm-40*	5mM Serine	23	45	ns	5mM Serine control
wt	*phgdh-1; tomm-40*	-	22	75		
wt	*phgdh-1; tomm-40*	5mM Serine	23	45	ns	*phgdh-1; tomm-40*
wt	*phgdh-1; tomm-40*	-	21	114		
wt	*phgdh-1; tomm-40*	5mM Serine	21	109	ns	*phgdh-1; tomm-40*
NR350 (Muscle specific RNAi)	control	-	27	35		
NR350 (Muscle specific RNAi)	*tomm-40*	-	30	172	****	NR350
NR350 (Muscle specific RNAi)	control	-	23	81		
NR350 (Muscle specific RNAi)	*tomm-40*	-	24	86	***	NR350
VP303 (Intestine specific RNAi)	control		23	190		
VP303 (Intestine specific RNAi)	*tomm-40*	-	24	100	**	VP303
VP303 (Intestine specific RNAi)	control	-	20	85		
VP303 (Intestine specific RNAi)	*tomm-40*	-	21	100	***	VP303
NR222 (Hypodermis specific RNAi)	control	-	23	102		

NR222 (Hypodermis specific RNAi)	*tomm-40*	-	23	98	ns	NR222
NR222 (Hypodermis specific RNAi)	control	-	23	57		
NR222 (Hypodermis specific RNAi)	*tomm-40*	-	23	255	ns	NR222
TU3401 (Neuron specific RNAi)	control	-	20	90		
TU3401 (Neuron specific RNAi)	*tomm-40*	-	20	97	ns	TU3401
TU3401 (Neuron specific RNAi)	control	-	23	126		
TU3401 (Neuron specific RNAi)	*tomm-40*	-	23	184	ns	TU3401
wt	control	-	17	115		
wt	control	D-glucose	16	220	****	control
wt	*tomm-40*	-	22	103	****	control
wt	*tomm-40*	D-glucose	18	192	****	*tomm-40*
wt	control	-	20	76		
wt	control	D-glucose	17	139	****	control
wt	*tomm-40*	-	22	108	****	control
wt	*tomm-40*	D-glucose	18	204	****	*tomm-40*
wt	control	-	21	78		
wt	*timm-23 ½*	-	25	64	****	control
wt	*ucr-1 ½*	-	24	65	****	control
wt	*timm-23 ½ ;ucr-1 ½*	-	29	54	****	*timm-23* and *ucr-1*
wt	*ucr-1 ½*	-	23	42		
wt	*timm-23 ½*	-	23	53		
wt	ucr-1 ½; *timm-23 ½*	-	25	60	****	*timm-23*
wt	control	-	21	106		
wt	*timm-23* 1/10	-	23	70	****	control
wt	cco-1 1/10	-	23	82	****	control
wt	*timm-23 9/10* ;cco-1 1/10	-	25	71	****	*timm-23 and cco-1*
wt	control	-	19	147		
wt	*timm-23* 9/10	-	21	120	****	control
wt	*cco-1* 1/10	-	21	157	****	control

wt	*timm-23* 9/10; *cco-1* 1/10	-	25	61	****	*timm-23 and cco-1*
wt	control	-	21	104		
wt	*timm-23* 9/10	-	23	70	****	control
wt	*atp-3* 1/10	-	27	50	****	control
wt	*timm-23* 9/10; atp-3 1/10	-	30	62	***	*timm-23 and atp-3*
wt	control	-	22	70		
wt	*timm-23*	-	26	92	****	control
haf-1(ok705)	control	-	22	22		
haf-1(ok705)	*timm-23*	-	30	30	****	*haf-1(ok705)*
dve-1(tm4803)	control	-	24	63		
dve-1(tm4803)	*timm-23*	-	36	65	****	*dve-1(tm4803)*
wt	control	-	20	113		
wt	*tomm-40*	-	26	125	****	control
dve-1(tm4803)	control	-	23	68		
dve-1(tm4803)	*tomm-40*	-	26	94	****	*dve-1(tm4803)*
wt	control	-	21	69		
wt	*tomm-40*	-	25	71	****	control
wt	*dve-1*	-	18	106		
wt	*dve-1;tomm-40*	-	19	95	****	*dve-1*
wt	*dve-1*	-	23	68		
wt	*dve-1;tomm-40*	-	26	94	****	*dve-1*
wt	control		22	67		
wt	*timm-23* from L4		24	117	*	wt
wt	*tomm-40* from L4		24	66	*	wt
wt	control	-	26	125		
wt	*timm-23* from L4	-	29	117	***	wt
wt	*tomm-40* from L4	-	26	94	**	wt
wt	OP50	0 μM DECA	17	60		
wt	OP50	5 μM DECA	17	59	*	wt
wt	OP50	10μM DECA	17	53	*	wt
wt	OP50	20μM DECA	18	49	****	wt
wt	HT115	0 μM DECA	20	60		
wt	HT115	5 μM DECA	20	59	ns	wt
wt	HT115	20μM DECA	20	49	**	wt
wt	control	-	24	117		
wt	*tomm-40*		27	87	****	wt
wt	*tomm-40* ½		24	131	ns	wt
wt	*hsp-60*		18	119	****	wt
wt	*hsp-60* ½		18	110	****	wt

wt	*hsp-60 ½;tomm-40 ½*		22	125	****	*hsp-60 ½*
wt	control		24	94		
wt	*tomm-40*		26	105	****	wt
wt	*tomm-40 ½*		24	144	ns	wt
wt	*hsp-60*		17	143	****	wt
wt	*hsp-60 ½*		17	137	****	wt
wt	*hsp-60 ½;tomm-40 ½*		24	138	****	*hsp-60 ½*
TU3401 (Neuron specific RNAi)	Control		18	220		
TU3401 (Neuron specific RNAi)	*cco-1*		18	317	ns	TU3401
TU3401 (Neuron specific RNAi)	*cco-1 1/10*		20	294	**	TU3401
TU3401 (Neuron specific RNAi)	Control		20	229		
TU3401 (Neuron specific RNAi)	*cco-1*		20	341	**	TU3401
TU3401 (Neuron specific RNAi)	*cco-1 1/10*		22	228	***	TU3401

Combination and dilution of different RNAi are denoted as *½, 1/10, 9/10.* Lifespan curves were statistically analyzed with the Log-rank (Mantel-Cox) test (**** denotes $P<0.0001$, *** denotes $P<0.001$, ** denotes $P<0.01$ and *denotes $P<0.05$)

Supplementary Table 3. The metabolites identified with significantly higher abundance in the tomm40 (RNAi) compared to the control worms at CT1 and CT2, based on the SAM method.

Metabolites with significant abundance increase	
**CT1**	**CT2**
1. CE_Un0002 (RT:15.648/QI:199//organic acid putatively)	1. glycerol
2. glycerol	2. glycine_effective
3. GABA	3. pyruvate
4. ethanolamine-phosphate	4. putrescine (4TMS) (Un_0129)
5. glycine_effective	5. serine_effective
6. 3-phosphoglycerate	6. lactate
7. myo-inositol	7. lysine (4TMS)
8. trehalose	8. threonine_effective
9.Un_0067 (P3458/a70/f_37// phospho-compound)	9. glycerol 3-phosphate
10. fumarate	10.Un_0063 (a_39/x_2/U_032// RT:20.913/QI:263)
11. beta-alanine	11. aminomalonic acid
12. threonine effective	12. cysteine NNO (4TMS)
13. aspartate (3TMS)	13. tryptophan_effective
14. serine (3TMS)	14.Un_0055 MeOx1 (A_116/f_26// RT: 27.611/QI:217// sugar)
15. lactate	15. Un_0017 (P1091/C_041)
16. putrescine (4TMS) (Un_0129)	16. myo-inositol
17. glutamate_effective	17. Unrev_289 (P3502// sugar phosphate)
18. lysine (4TMS)	18. CE_Un0007 (RT:28.152/QI:117)
19. Un_0088 (K_47/A_077// RT:22.282/QI:188)	19.Un_0102 (P3188/U_062// fatty acid putatively)
20. malate	20. sorbitol
21. Un_0017 (P1091/C_041)	21. A_052 (RT:17.309/QI:298)
22. vitamin B5 (Un_0060/P3075/a_68/unknown no 114)	22. trehalose
FDR-median = 0%	23. Un_0126 (A_170// RT:34.244/QI:339)
23. threonate	24. threonate
24. A_052 (RT:17.309/QI:298)	25. malate
25. 2-hydroxyglutaric acid	***FDR-median = 0%***
26. glycerol monostearate	26. CE_Un0002 (RT:15.648/QI:199//organic acid putatively)
27. urea (2TMS)	27. vitamin B5 (Un_0060/P3075/a_68/unknown no 114)
28. isoleucine (2TMS)	28. glucose_total
29. citrate	29. glycerol monostearate
30. Un_0136 (P3960// fatty acid putatively)	30. Un_0136 (P3960// fatty acid putatively)
31. Unrev_289 (P3502// sugar phosphate)	31. CE_Un0008 (RT:29.068/QI:129)
32. aminomalonic acid (3TMS)	32. CE_Un0004 (RT:24.276/QI:266)
33. CE_Un0007 (RT:28.152/QI:117)	33. benzoate
34. phosphate (4TMS)	34. myristate

35. sorbitol (6TMS)	35. octadecanoic acid
36. glucose-6P MeOx1	36. alanine_effective
FDR-median = 0.329 %	***FDR-median = 0.486 %***

The results for each collection time are separated into two groups: the first includes the positively significant metabolites for the smallest significance threshold for which the FDR-median is 0 and the second, the additional metabolites identified as positively significant for the smallest possible significant threshold for these datasets based on SAM, still corresponding to a very small FDR-median. The corresponding SAM curves are shown in Supplementary Figs 7c and 8c for CT1 and CT2, respectively. The relative abundances of these metabolites for CT1 and CT2 are shown in the heat maps of Fig. 5a and b.

Blots from Supplementary Figure 3e

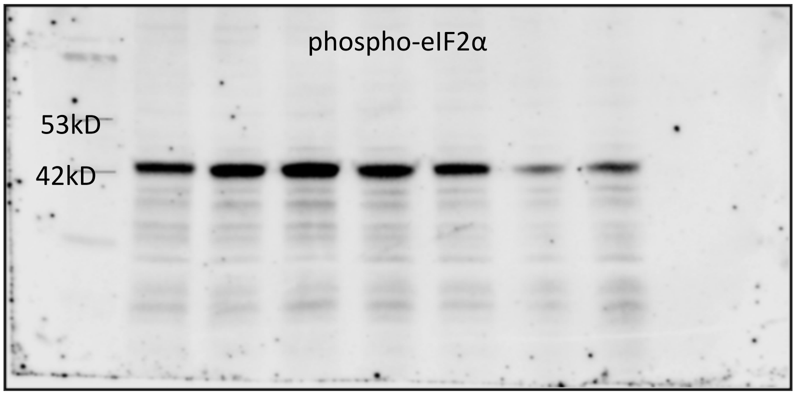

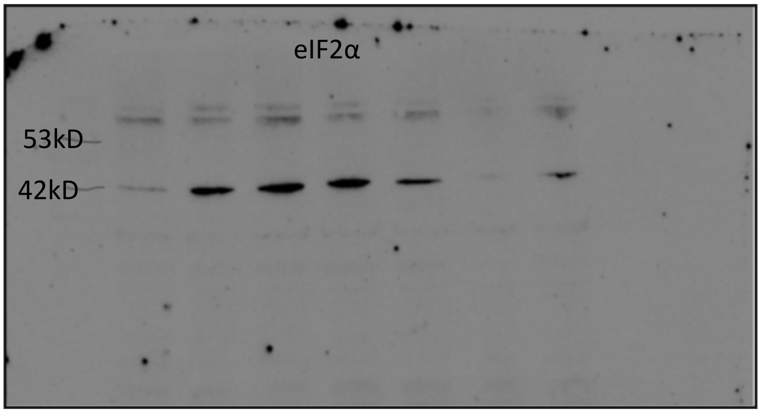

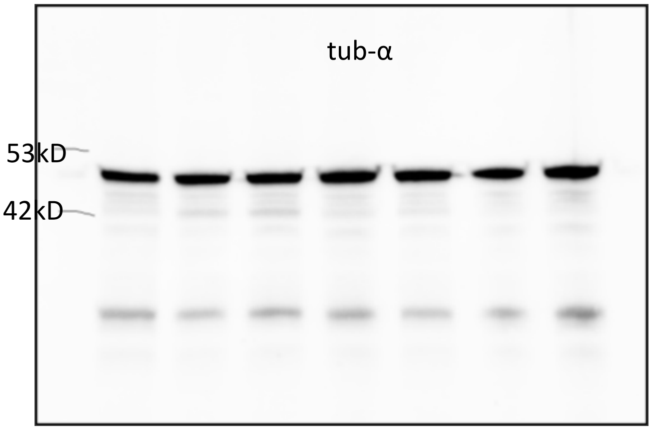

Supplement: Supplementary file 1 — Supplementary Information [file 41467_2022_28272_MOESM1_ESM.pdf]
