# Supplementary material for: Mitochondrial protein import determines lifespan through metabolic reprogramming and de novo serine biosynthesis
Source: Nat Commun. 2022 Feb 3;13:651. doi: 10.1038/s41467-022-28272-1 (PMC8814026; doi:10.1038/s41467-022-28272-1)
Supplement: Supplementary file 3 — Description of Additional Supplementary Files [file 41467_2022_28272_MOESM3_ESM.docx]

**DESCRIPTION OF ADDITIONAL SUPPLEMENTARY FILES**

**Mitochondrial protein import determines lifespan through metabolic reprogramming and *de novo* serine biosynthesis**

**Eirini Lionaki^1,α^*, Ilias Gkikas^1,2,α^, Ioanna Daskalaki^1,2^, Maria-Konstantina Ioannidi^3,4^, Maria I. Klapa^3^ & Nektarios Tavernarakis^1,5^***

^1^Institute of Molecular Biology and Biotechnology, Foundation for Research and Technology - Hellas, ^2^Department of Biology, School of Sciences and Engineering, University of Crete, ^3^Metabolic Engineering and Systems Biology Laboratory, Institute of Chemical Engineering Sciences, Foundation for Research and Technology-Hellas (FORTH/ICE-HT), Patras, Greece; ^4^Department of Biology, University of Patras, Patras, Greece; ^5^Department of Basic Sciences, Faculty of Medicine, University of Crete, Heraklion 71110, Crete, Greece.

*Correspondence and requests for materials should be addressed to E.L. (e-mail: lionaki@imbb.forth.gr), or to N.T. (e-mail: tavernarakis@imbb.forth.gr).

**File name: Supplementary Data 1**

**Description: Sheet A**. The Raw GC-MS Metabolomic Dataset (Peak Areas) considered in the analyses. **Sheet B**. The normalized mean GC-MS metabolic profiles considered in the analyses; the profiles have been transformed -when needed - to correspond to 100 mg of worm pellet. **Sheet C**. The % fraction of each metabolite in the total quantified RPA.
