# Supplementary material for: Mitochondrial protein import determines lifespan through metabolic reprogramming and de novo serine biosynthesis
Source: Nat Commun. 2022 Feb 3;13:651. doi: 10.1038/s41467-022-28272-1 (PMC8814026; doi:10.1038/s41467-022-28272-1)
Supplement: Supplementary file 5 — Reporting Summary [file 41467_2022_28272_MOESM5_ESM.pdf]

nature research

Corresponding author(s): Nektarios Tavernarakis, Eirini Lionaki

Last updated by author(s): Jan 12, 2022

Reporting Summary

Nature Research wishes to improve the reproducibility of the work that we publish. This form provides structure for consistency and transparency in reporting. For further information on Nature Research policies, see Authors & Referees and the Editorial Policy Checklist .

Statistics

For all statistical analyses, confirm that the following items are present in the figure legend, table legend, main text, or Methods section.

n/a	Confirmed	
☐	☒	The exact sample size (*n*) for each experimental group/condition, given as a discrete number and unit of measurement
☐	☒	A statement on whether measurements were taken from distinct samples or whether the same sample was measured repeatedly
☐	☒	The statistical test(s) used AND whether they are one- or two-sided<br>*Only common tests should be described solely by name; describe more complex techniques in the Methods section.*
☒	☐	A description of all covariates tested
☐	☒	A description of any assumptions or corrections, such as tests of normality and adjustment for multiple comparisons
☐	☒	A full description of the statistical parameters including central tendency (e.g. means) or other basic estimates (e.g. regression coefficient) AND variation (e.g. standard deviation) or associated estimates of uncertainty (e.g. confidence intervals)
☐	☒	For null hypothesis testing, the test statistic (e.g. *F*, *t*, *r*) with confidence intervals, effect sizes, degrees of freedom and *P* value noted<br>*Give P values as exact values whenever suitable.*
☒	☐	For Bayesian analysis, information on the choice of priors and Markov chain Monte Carlo settings
☒	☐	For hierarchical and complex designs, identification of the appropriate level for tests and full reporting of outcomes
☒	☐	Estimates of effect sizes (e.g. Cohen's *d*, Pearson's *r*), indicating how they were calculated

Our web collection on statistics for biologists contains articles on many of the points above.

Software and code

Policy information about availability of computer code

Data collection	For the collection of the metabolomic data, the MS Workstation software v, 6.4 was used for reading out the MS-reconstructed chromatograms, identifying peaks and quantifying peak areas
Data analysis	Image analysis was performed with ImageJ bundled with 64-bit Java 1.8.0_172, Statistical analysis was performed with GraphPad Prism 8.0. For metabolomic data identfication, commercial NIST library v.2.0 as incorporated in the MS Workstation software, and in-house MESBL peak library of >900 reviewed peaks (Maga-Nteve and Klapa, 2016) were used, for metabolomic data normalization and filtering, in-house M-IOLITE software was used (http://miolite2.iceht.forth.gr) and multivariate statistical analysis, the TM4/MeV omic analysis software v 4.9.0 were used.

There are no custom algorithms or software that are central to the research. For manuscripts utilizing custom algorithms or software that are central to the research but not yet described in published literature, software must be made available to editors/reviewers. We strongly encourage code deposition in a community repository (e.g. GitHub). See the Nature Research guidelines for submitting code & software for further information.

Data

Policy information about availability of data

All manuscripts must include a data availability statement. This statement should provide the following information, where applicable:

- Raw data from all figures and supplementary figures are included in the source data file. The raw data from the metabolomics analysis are provided as a separate sheet in the supplementary Data File 1. The respective chromatographs are accessible via this link: https://tavernarakislab.gr/publications/lionaki_et_al.zip - There are no restrictions for data availability

The authors declare that all data supporting the findings of this study are available within the paper, its supplementary information files and the link

Field-specific reporting

Please select the one below that is the best fit for your research. If you are not sure, read the appropriate sections before making your selection.

☒ Life sciences ☐ Behavioural & social sciences ☐ Ecological, evolutionary & environmental sciences

For a reference copy of the document with all sections, see nature.com/documents/nr-reporting-summary-flat.pdf

Life sciences study design

All studies must disclose on these points even when the disclosure is negative.

Sample size

For C. elegans lifespan experiments used more than 50 animals per individual experiment.

Fluorescence imaging experiments at least 20 animals were used per condition
In metabolomics, two biologically independent replicates (pooled pellet of ~100µg from multiple animals) were analyzed per condition and the metabolic profile of each replicate was analyzed at least thrice - the full list of replicates is shown in Supplementary Datafile 1A). The analysis was carried out twice at two independent collection times.

Data exclusions

No data exclusion

Replication

Lifespan experiments were performed at least 2 times with similar results. Imaging analysis was performed at least 3 times with similar results. Metabolic profiling was carried out at two independent collection times and periods in the laboratory. A standardized profile acquisition protocol was used (Papadimitropoulos et al., 2018) and the conditions of the procedure were validated as the same between the two experiments (collection times) based on a GC-MS metabolomic data validation criterion described in Kanani and Klapa, 2007; Papadimitropoulos et al. 2018 - validation estimates shown in Suppl. Datafile 1

Randomization

Animals were synchronized by hypochloride treatment. ~1000 eggs were placed on culture plates and reared until first day of adulthood. Then, adult animals were randomly selected by each culture plate. All animals required for the analysis were in close proximity and picked from a specific area of the plate without selection based on body size or fitness.

Blinding

Blinding to group allocation was not possible as MitoMISS animals have an obvious phenotype (smaller animals). All experiments with objective measurements (such as microscopy and lifespan assays) were also performed by other members of the laboratory.

Reporting for specific materials, systems and methods

We require information from authors about some types of materials, experimental systems and methods used in many studies. Here, indicate whether each material, system or method listed is relevant to your study. If you are not sure if a list item applies to your research, read the appropriate section before selecting a response.

Materials & experimental systems

n/a	Involved in the study
☐	☒ Antibodies
☒	☐ Eukaryotic cell lines
☒	☐ Palaeontology
☐	☒ Animals and other organisms
☒	☐ Human research participants
☒	☐ Clinical data

Methods

n/a	Involved in the study
☒	☐ ChIP-seq
☒	☐ Flow cytometry
☒	☐ MRI-based neuroimaging

Antibodies

Antibodies used

anti-ATP5A (Abcam, ab14748), anti-eiF2 subunit 1α (Cell Signaling, 9722), anti-phospho-eiF2α (Cell Signaling, 119A11), anti-alpha-tubulin (DSHB 12G10)
All antibodies were used in 1/1000 dilution except for a-α-tubulin which was used in 1/5000 dilution.

Validation

anti-ATP5A has a broad species specificity and has been validated on *C. elegans* samples in 25773600. total and phospho-eIF2α antibodies have a broad species specificity and crossreact with *C.elegans* samples according to manufacturer instruction. Also validated in 26677221 and in our study with eIF2α RNAi samples. a-alpha-tubulin antibody crossreact with *C.elegans* samples according to manufacturer instruction and validated in our study.

Animals and other organisms

Policy information about studies involving animals; ARRIVE guidelines recommended for reporting animal research

Laboratory animals

Caenorhabditis elegans hermaphrodites at day 1 of adulthood were used unless otherwise specified

Wild animals

No wild animals were used in this study

Field-collected samples

No field-collected animals were used in this study

Ethics oversight

No ethical approval is required for this study

Note that full information on the approval of the study protocol must also be provided in the manuscript.
